# Breeding habitats, bionomics and phylogenetic analysis of *Aedes aegypti* and first detection of *Culiseta longiareolata*, and *Ae*. *hirsutus* in Somali Region, eastern Ethiopia

**DOI:** 10.1371/journal.pone.0296406

**Published:** 2024-01-02

**Authors:** Solomon Yared, Araya Gebressilasie, Amha Worku, Abas Mohammed, Isuru Gunarathna, Dhivya Rajamanickam, Elizabeth Waymire, Meshesha Balkew, Tamar E. Carter

**Affiliations:** 1 Department of Biology, Jigjiga University, Jigjiga, Ethiopia; 2 Department of Zoological Sciences, Addis Ababa University, Addis Ababa, Ethiopia; 3 Department of Biology, College of Arts and Sciences, Baylor University, Waco, TX, United States of America; 4 Abt Associates, PMI VectorLink Ethiopia Project, Addis Ababa, Ethiopia; Uppsala University: Uppsala Universitet, SWEDEN

## Abstract

**Introduction:**

Arboviral diseases, such as dengue, chikungunya, yellow fever, and Zika, are caused by viruses that are transmitted to humans through mosquito bites. However, the status of arbovirus vectors in eastern Ethiopia is unknown. The aim of this study was to investigate distribution, breeding habitat, bionomics and phylogenetic relationship of *Aedes aegypti* mosquito species in Somali Regional State, Eastern Ethiopia.

**Methods:**

Entomological surveys were conducted in four sites including Jigjiga, Degehabur, Kebridehar and Godey in 2018 (October to December) to study the distribution of *Ae*. *aegypti* and with a follow-up collection in 2020 (July-December). In addition, an investigation into the seasonality and bionomics of *Ae*. *aegypti* was conducted in 2021 (January-April) in Kebridehar town. Adult mosquitoes were collected from indoor and outdoor locations using CDC light traps (LTs), pyrethrum spray collection (PSCs), and aspirators. Larvae and pupae were also collected from a total of 169 water-holding containers using a dipper between October and November 2020 (rainy season) in Kebridehar town. The species identification of wild caught and reared adults was conducted using a taxonomic key. In addition, species identification using mitochondrial and nuclear genes maximum likelihood-based phylogenetic analysis was performed.

**Results:**

In the 2018 collection, *Ae*. *aegypti* was found in all study sites (Jigjiga, Degahabour, Kebridehar and Godey). In the 2020–2021 collection, a total of 470 (Female = 341, Male = 129) wild caught adult *Ae*. *aegypti* mosquitoes were collected, mostly during the rainy season with the highest frequency in November (n = 177) while the lowest abundance was in the dry season (n = 14) for both February and March. The majority of *Ae*. *aegypt* were caught using PSC (n = 365) followed by CDC LT (n = 102) and least were collected by aspirator from an animal shelter (n = 3). *Aedes aegypti* larval density was highest in tires (0.97 larvae per dip) followed by cemented cisterns (0.73 larvae per dip) and the Relative Breeding Index (RBI) was 0.87 and Container Index (CI) was 0.56. Genetic analysis of *ITS2* and *COI* revealed one and 18 haplotypes, respectively and phylogenetic analysis confirmed species identification. The 2022 collection revealed no *Ae*. *aegpti*, but two previously uncharacterized species to that region. Phylogenetic analysis of these two species revealed their identities as *Ae*. *hirsutus* and *Culiseta longiareolata*.

**Conclusion:**

Data from our study indicate that, *Ae*. *aegypti* is present both during the wet and dry seasons due to the availability of breeding habitats, including water containers like cemented cisterns, tires, barrels, and plastic containers. This study emphasizes the necessity of establishing a national entomological surveillance program for *Aedes* in Somali region.

## Introduction

Arboviral diseases such as dengue, chikungunya, yellow fever, and Zika, are caused by viruses that are spread to people by the bite of an infected arthropod, mosquitoes. However, these diseases are considered neglected diseases and low priority has been given to their research in past decades. Due to the urbanization, globalization, and increased human mobility, unprecedented emergence of arboviral disease epidemics have been increased over time, and thus the need to carry out surveillance and research related to the arboviral diseases, and their vectors has risen [1].

Mosquito-borne viral infections such as yellow fever (YF), dengue fever (DF), and chikungunya (CHIK) are occurring frequently in some areas of Ethiopia. In recent years, outbreaks of DF occurred in the eastern, southeastern, southern and northwest parts of the country [2–5]. Several factors are related to the increase of DF incidence in Ethiopia. Among the most important ones are uncontrolled urbanization and the absence of standardized public services, such as water supply, sewage and waste disposal [3].

*Aedes aegypti* and *Ae*. *albopictus* are the known vectors of dengue virus (DENV) and other arboviruses including chikungunya virus (CHIKV) and zika virus (ZV) [6]. These species are highly efficient vectors of arboviruses and live in close proximity to humans [7]. *Ae*. *africanus* and *Ae*. *luteocephalus* also act as potential vectors in Africa [8]. *Ae*. *aegypti* is uniquely adapted to a close association with humans, which facilitates efficient virus transmission. Immature forms of the mosquitoes develop primarily in man-made containers in and around the homes of people [9, 10]. It is a highly anthropophilic, endophilic and endophagic day-biting species that rests inside houses where females feed frequently and preferentially on human blood [11]. These behavioral traits enhance human–mosquito contacts and dengue virus attack rates to be very high [12].

In 2017 an outbreak of dengue fever was reported in Kebridehar Town in the Somali region of Ethiopia [13]. In this study, a total of 101 cases occurred including five with severe dengue and one death were reported from 10 villages of the town. Despite the public health significance and the recent occurrences of dengue fever in this particular urban area, no detailed entomological studies to determine the breeding habitats, bionomics, and population genetic structure of *Ae*. *aegypti* were conducted. Therefore, the current study was designed to investigate the distribution, breeding habitats, bionomics, and genetic diversity of the of *Aedes aegypti* in Somali Region, Ethiopia.

## Material and methods

### Mosquito collection

**Fig 1:**
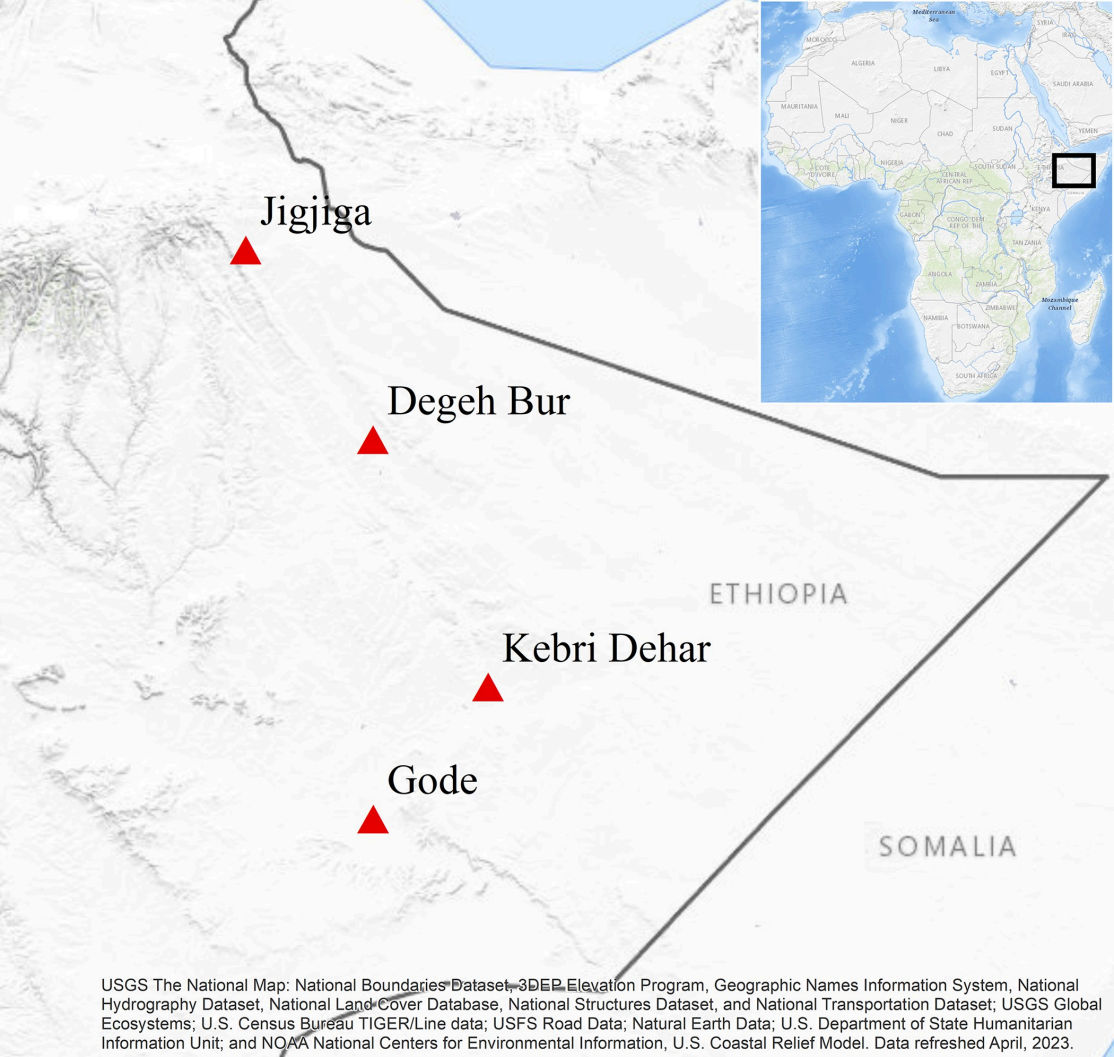
Map of study sites.

Adult mosquitoes were collected from indoor and outdoor using Centers for Disease Control miniature light traps (John W. Hock, Gainesville, FL) (CDC LT), pyrethrum spray collection (PSC) techniques and mouth aspirator from animal shelters (Cattle and Goat) in four urban areas of Somali region in 2018, 2020 and 2021. In 2018 (October to December), adult mosquito collections were carried out in four sites including Jigjiga, Degahabur, Kebridehar and Godey (Fig 1). In addition, mosquitoes were also collected in Kebridehar from July-December of 2020 and January-April of 2021. Finally, a follow-up survey was conducted in Godey and Jigjiga 2022. Jigjiga University research review committee approved the study and a support letter was obtained from Somali Regional health bureau for mosquito house collection. Verbal informed consent was sought from participants prior to enrolling their house in the study.

### CDC light trapping (CDC LT)

Twenty houses were selected for indoor and outdoor light trap catches from the same villages of Kebridehar town based on their relative location from the breeding site in a similar way to houses selected. Mosquitoes were collected from both indoor and outdoor locations from 6:00 pm to 6:00 am from each selected house using standard CDC LT. Traps were hung from the ceiling or from roof supports at the foot end of the bed where people sleep at night and the body of the trap was suspended about 1.5 meters from the floor.

Collection bags were attached to the trap by stretching the open end of the bag over the bottom rim of the trap and a label marked with the date and the site number was placed inside the collection bag. Collection bags were retrieved from traps in each house in the morning from 8:00 am to 9:00 am by entomological assistants.

### Pyrethrum Spray Sheet collections (PSC)

Indoor resting *Aedes* sp. were sampled in the morning (6:00 to 8:00 Am) from 20 randomly selected houses in each study area by the application of pyrethrum spray catches. In PSC monthly mosquito collections were done in the same houses throughout the study period. Before spraying in each house, all food items were removed; the windows and door openings and eaves were filled with pieces of cloth, and the floor was covered with white cloth sheets. After the door was closed, the pyrethrum aerosol was sprayed for about three to five minutes. After ten minutes of spraying, the knockdown *Aedes* mosquitoes were then collected from the white sheets using fine forceps and placed in Petri dish for later processing and identification. Adult female *Aedes* mosquitoes were categorized into unfed, fresh fed, semi-gravid, and gravid according to their abdominal status under a dissecting microscope.

### Mouth aspirator collection

*Aedes* mosquitoes were collected using mouth aspirator in the early evening and morning from an animal shelter, and other locations.

### Processing of adult mosquitoes

Every morning during the collection period, mosquitoes captured in CDC LT, PSC, and aspirator from were transported to a field station laboratory and mosquitoes were transferred to test tubes. They were then knocked down using chloroform and emptied on petri dishes for sorting into distinct categories (genera, sex, and abdominal status) under a dissecting microscope. The adult mosquitoes were identified morphologically using a standard key [14].

### Larval and pupae collection

**Fig 2:**
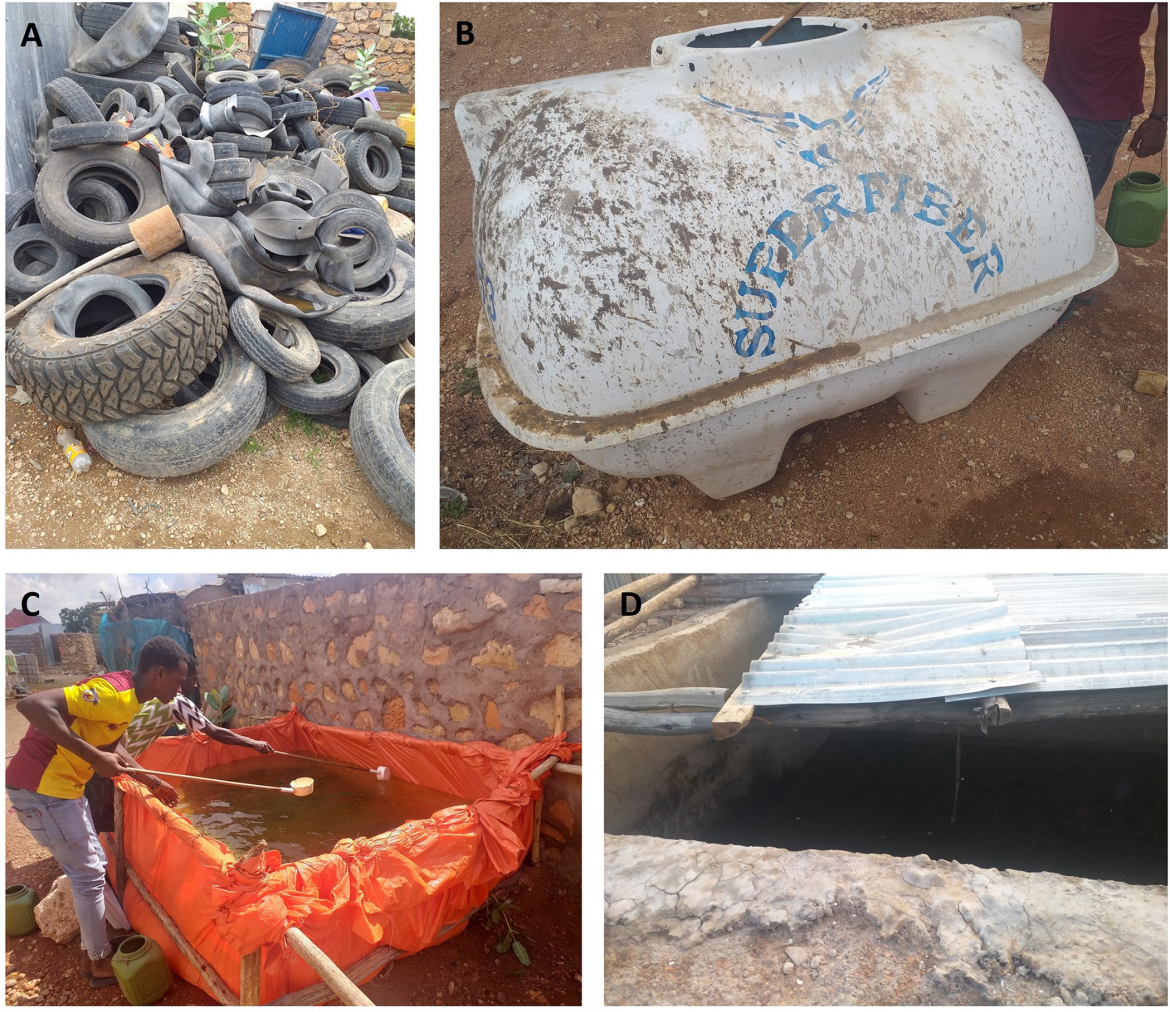
Potential Breeding habitats of *Aedes aegypti* at study sites including A) abandoned tires, B) water tanks, C) cisterns, and D) plastic containers.

A house-to-house mosquito breeding habitat survey was conducted in Kebridehar Town. Larvae and pupae were also collected from a total of 169 water-holding containers using a dipper between October and November 2020 in Kebridehar (rainy season) (Fig 2). Some other larvae and pupae mosquito specimens were collected in Jigjiga June 2022 that were morphologically similar to *Aedes* but not initially identified (S1 and S2 Figs).

Larvae and pupae of mosquitoes were collected from breeding habitats such as cemented cisterns, water containers, discarded tires, plastic containers and barrels. The presence of immature stages of mosquitoes was visually evaluated in all water-holding containers present in the premises of the houses. The number and type of containers inspected were recorded, including information on which are present or absent immature stages of mosquitoes. During sampling, 20 dips were made using a standard dipper from each positive breeding habitat. The collected immature stage was transported to a field laboratory. All collected larvae and pupae were reared in the laboratory until adults emerged, and all adults that emerged from the pupae were collected and stored in vials and carefully classified by species according to the pattern of white bands, using a dissecting microscope and identification keys [14].

### Larval indices analysis

The larval survey data were calculated and analyzed in terms of different larval survey techniques like Container Index (CI), and Breteau Index (BI). The calculation of larval indices is based on the following mathematical formulae:

$$Container\;Index(CI)=\frac{Number\;of\;Positive\;containers\;infested}{Total\;number\;of\;containers\;inspected}X100$$

$$Breteau\;Index(BI)=\frac{Number\;of\;positive\;containers}{Total\;number\;of\;houses\;inspected}X100$$

### DNA extraction and molecular identification of mosquitoes

Molecular species confirmation was performed on a subset of specimens collected from Kebridehar, Degehabur, and Jigjiga. DNA was extracted from the abdomens selected at random, using the DNeasy Blood and Tissue Kit (Qiagen, Valencia, CA). Polymerase chain reaction (PCR) was performed for each individual mosquito, targeting the nuclear internal transcribed spacer 2 (ITS2) region. The reagent components and concentrations for the PCR assays were 1X Promega HotStart Master Mix (Promega, Madison, WI) and 0.5 mM for both primers, plus 1 μL of isolated DNA template. Primers used in these ITS2 reactions were 5.8SB-5’ATG CTT AAA TTT AGG GGG TAG TC-3’ and 28SB 5’-ATG CTT AAA TTT AGG GGG TAG TC-3’. The PCR protocol consisted of 95°C for 2 min, 31 cycles of 95°C for 1 min, 49°C for 30 sec, and 72°C for 1 min, followed by an extension step of 72°C for 5 min. The cytochrome *c* oxidase subunit 1 (COI) was amplified to determine any polymorphisms. Primers used in this reaction included LCO1490F 5’-GGTCAACAAATCATAAAGATATTGG-3’ and HCO2198R 5ʹ-TAAACTTCAGGGTGACCAAAAAATCA-3ʹ. A negative control of no DNA template was used to ensure no contamination of PCR reagents. The reagent components and final concentrations for the PCR assays were 1X Promega HotStart Master Mix (Promega, Madison, WI) and 0.5 mM for both primers, plus 1 uL of isolated DNA template. For COI, the PCR protocol was as follows: 95°C for 1 min, 31 cycles of 95°C for 30 sec, 48°C for 30 sec, and 72°C for 1 min, followed by an extension step of 72°C for 10 min. Amplicons were sequenced using Sanger technology with ABI BigDye^TM^ Terminator chemistry (Thermofisher, Santa Clara, CA) according to manufacturer recommendations and run on a 3170xl Genetic Analyzer (Thermo Fisher, Santa Clara, CA) through Eurofins Genomics (Louisville, USA).

### Sequence and phylogenetic analysis

Sequences were cleaned and analyzed using CodonCode (CodonCode Corporation, Centerville, MA, USA). ITS2 and COI sequences were submitted as queries to the National Center for Biotechnology Information’s (NCBI) Basic Local Alignment Search Tool (BLAST) against the nucleotide collection under standard parameters (100 max target sequences, expect threshold 0.05, word size 28, optimized for highly similar sequences, not specific to any organism).

Alignments were created with MAFFT version 7 (Katoh K, Rozewicki J, Yamada KD. MAFFT online service: multiple sequence alignment, interactive sequence choice and visualization. Brief Bioinform. 2019;20:1160–6.) using Codon Code version 8.01 (CodonCode Corporation, Dedham, MA, USA) and Mesquite version 3.61 (Wane P. Maddison DRM: Mesquite: a modular system for evolutionary analysis. 2021. http://www.mesquiteproject.org.). Phylogenetic associations between sequences were estimated using a maximum likelihood approach with RAxML (Stamatakis A. RAxML-VI-HPC: maximum likelihood-based phylogenetic analyses with thousands of taxa and mixed models. Bioinformatics. 2006; 22:2688–90). The GTRGAMMA option that utilizes the GTR model of nucleotide substitution with gamma model of rate of heterogeneity was used. One thousand runs were completed using the maximum likelihood criteria with rapid bootstrap analysis. The RAxML output was viewed in FigTree, with a root at the out group, and a phylogenetic tree image was made.

## Results

In total, 543 *Ae*. *aegypti* were captured from urban areas during various collection years (Table 1). During the 2018 collection, *Ae*. *aegypti* was recorded in all study sites and the highest number of *Ae*. *aegypti* was found in the town of Kebridehar (n = 35), followed by Godey (n = 19), Degehabur (n = 16), and Jigjiga (n = 3). Additionally, a total of 470 *Ae*. *aegypti* were captured in Kebridehar town during the 2020/2021 collections.

**Table 1:** Summary of the number of *Aedes aegypti* collected from 2018–2021. Dash signifies no survey conducted.

Sites	October–December, 2018	July–December, 2020	January–April, 2021	February–June, 2022	Total
Jigjiga	3	-	-	0	3
Degehabur	16	-	-	0	16
Kebridehar	35	382	88	-	505
Godey	19	-	-	0	19
Total	73	382	88	0	543

**Fig 3:**
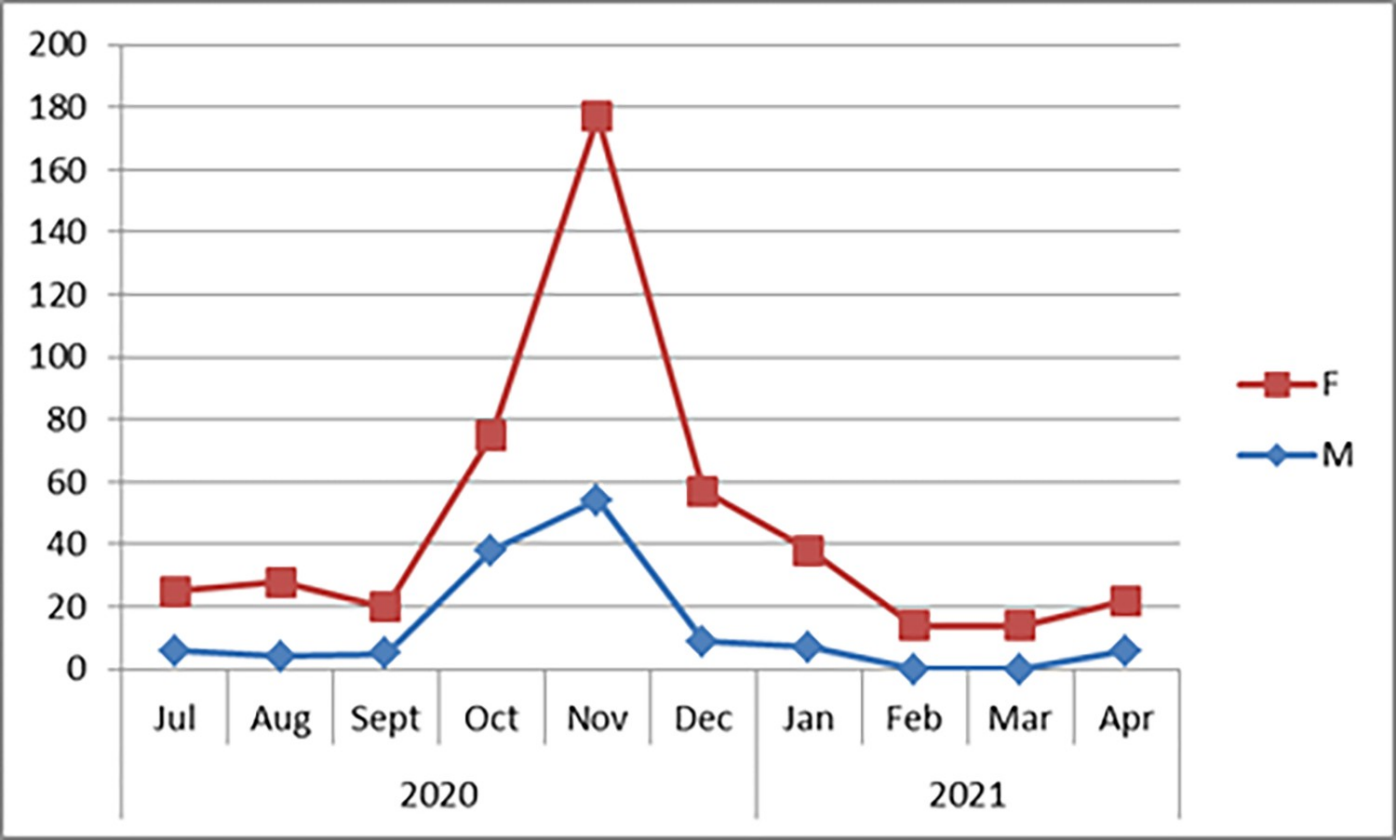
Monthly number of *Aedes aegypti* collected from Kebridehar town from CDC light trap, PSC, and aspirator.

**Fig 4:**
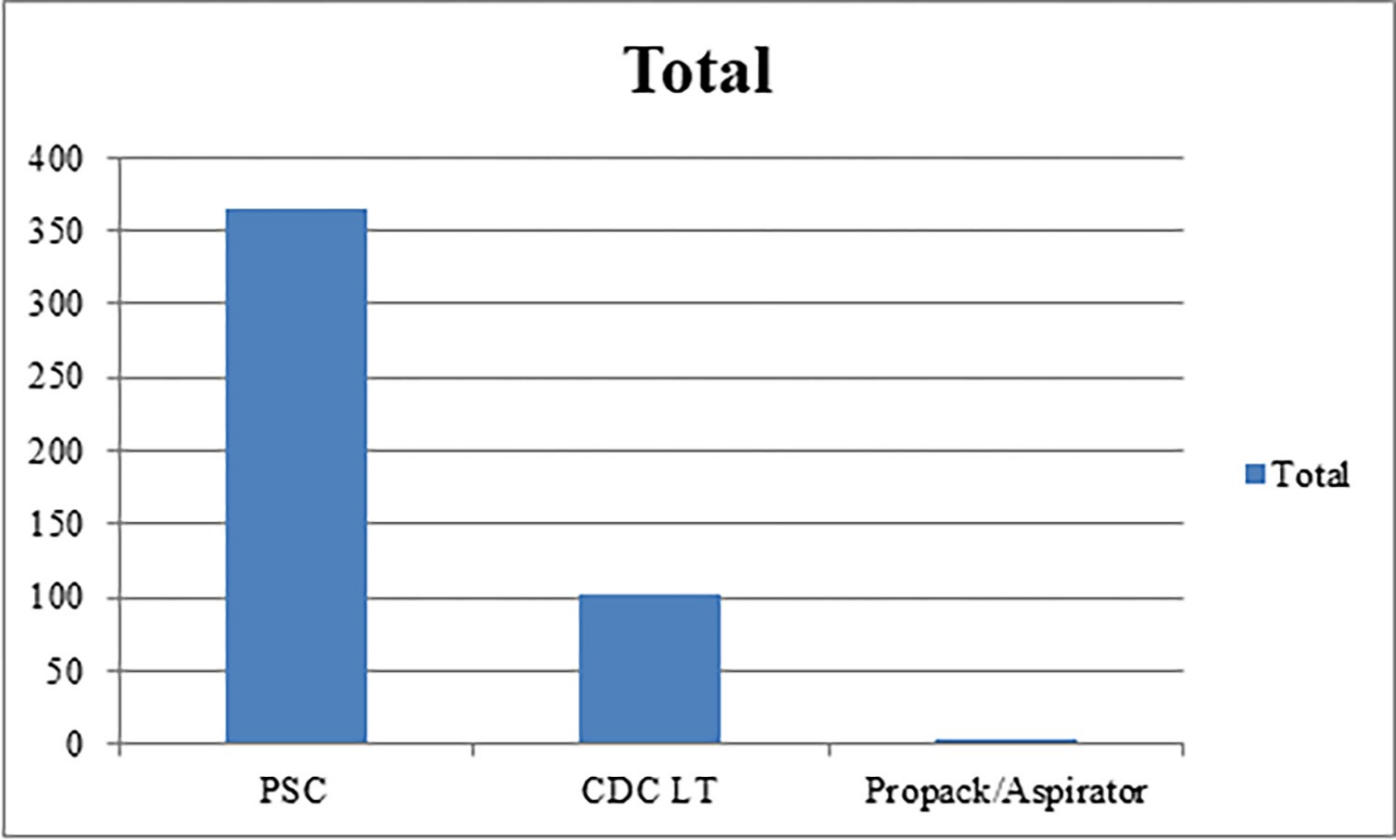
Total number of *Aedes aegypti* collected.

**Fig 5:**
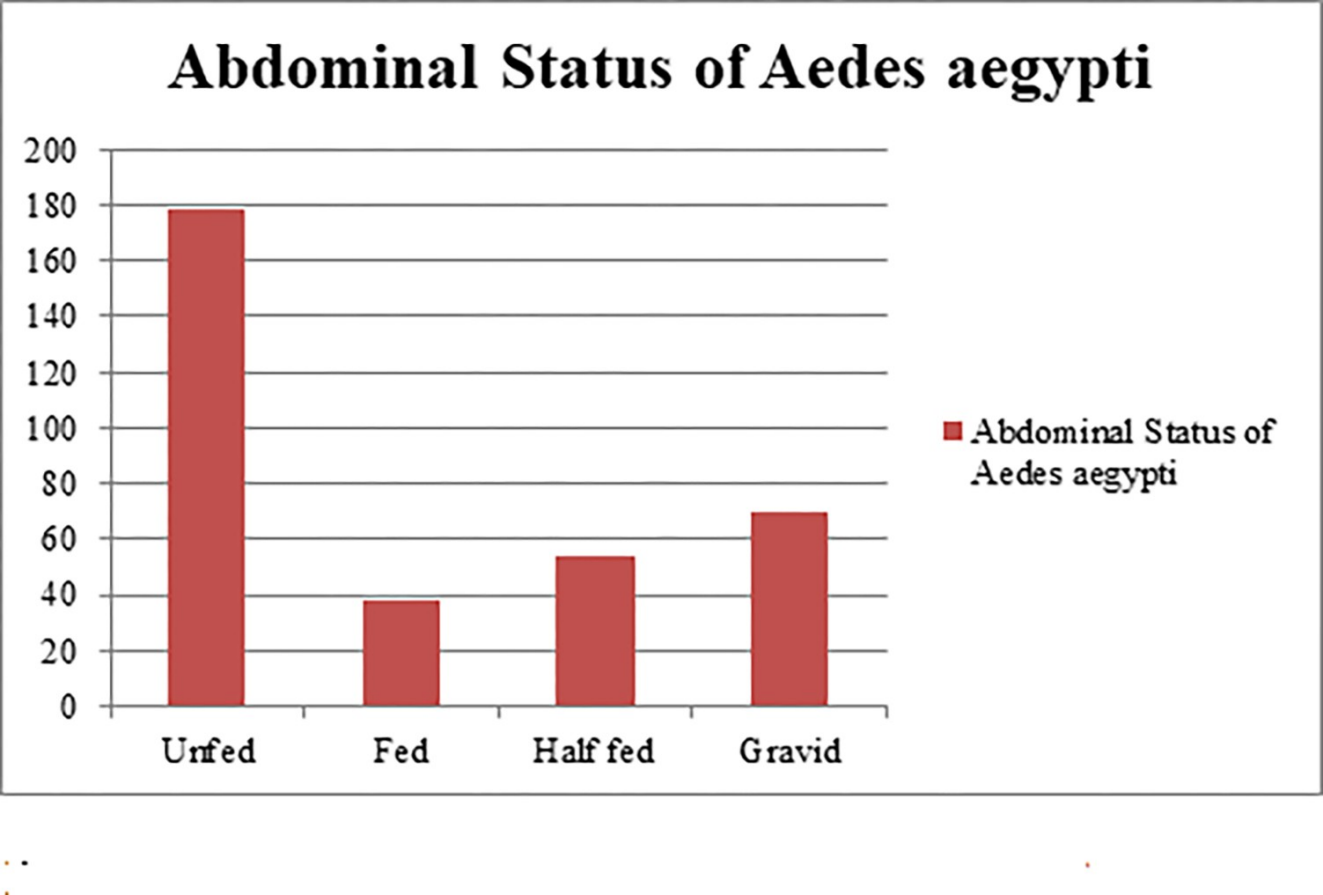
Abdominal status of female *Aedes aegypti*.

During the 2020/2021 seasonal collection, a total of 470 (Female = 341, Male = 129) wild caught adult *Ae*. *aegypti* mosquitoes were collected from Kebridehar Town of Somali region. A total of 309 *Ae*. *aegypti* were recorded in the wet season [Nov (n = 177), Oct (n = 75), and Dec (n = 57)] and 28 *Ae*. *aegypti* were recorded in dry season [Feb (n = 14) and March (n = 14)] (Fig 3). Most *Ae*. *aegypti* were caught using PSC (n = 365) followed by CDC LT (n = 102) and aspirator from an animal shelter (n = 3). *Aedes aegypti* were collected from outdoor and indoor in Kebridehar town; more were caught from indoors using PSC than aspirator and CDC LTs during the collection time (Fig 4). Analysis of the abdominal section revealed, unfed (n = 179), gravid (n = 70), half fed (n = 54), and fresh fed (n = 38) specimens (Fig 5).

### Larval density

In total 1419 larvae of *Aedes* mosquitoes and 329 pupae were sampled from cemented cisterns, tires, plastic containers, and barrels (Table 2). *Aedes* larval density was highest in tires (0.97 larvae per dip) followed by cemented cisterns (0.73 larvae per dip). The Relative Breeding Index (RBI) was 0.87 and Container Index (CI) was 0.56. Container locations were also observed during the entomological assessment, along with water status and types. The majority of the water holding containers that were positive for larvae/pupae were placed outside within an average 10-meter radius of the houses.

**Table 2:** Larval density, adults emerged from larvae and breeding site indices for each breeding site container type in Kebridehar.

	Cisterns	Tires	Plastic containers	Barrels	Water pool	Overall total* or average**
Number of containers inspected	136	17	10	4	2	169*
Number of positive containers for *Ae*. *aegypti*	72	13	6	4	0	95*
RBI (Relative Breeding Index) for *Ae*. *aegypti*^ǂ^	0.9	0.8	0.6	1	0	0.87**
Habitat Index (Container Index) for *Ae*. *aegypti*	0.53	0.76	0.6	1	0	0.56**
Total larvae *Aedes*	1051	251	61	56	0	1419*
Total larvae Culex and Anopheles	1164	312	99	56	0	1631
Pupae	231	81	2	15	0	329*
Total number of dips	1440	260	120	80	0	1900
Larval density per dip	0.73	0.97	0.5	0.7	0	0.75**
Number and species of *Ae*. *aegypti* reared from larvae and pupae	1182	302	62	69	0	1615*

ǂ RBI relative breeding index (number of habitats positive for *Ae*. *aegypti* divided by the total number of habitats positive for any mosquito)

### Phylogenetic analysis of specimens

**Fig 6:**
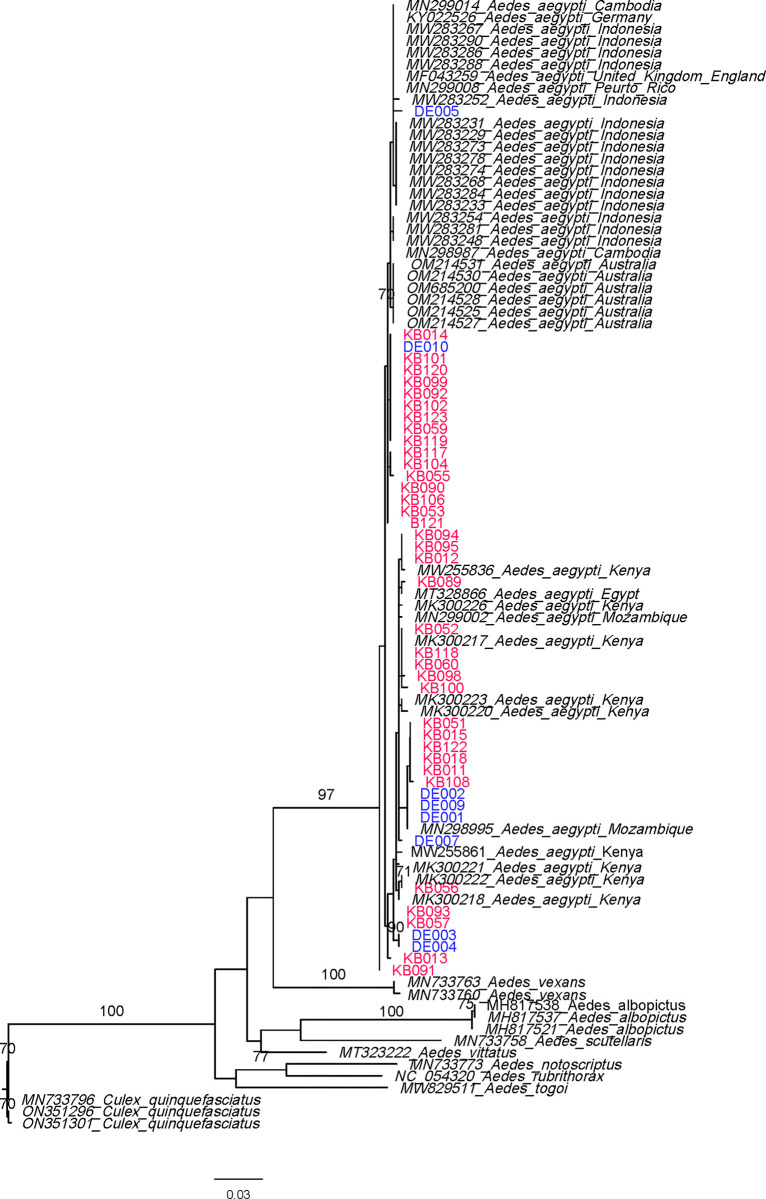
Maximum likelihood phylogenetic tree of *Aedes aegypti COI*. Ethiopian sequences and *Aedes aegypti* reference sequence are indicated by bracket. Ethiopian sequences are in color (red = Kebridehar, blue = Degehabur). Bootstrap value are based on 1000 replicates. Only bootstraps above 70 are shown.

For the morphologically identified *Ae*. *aegypti*, *a* single haplotype was observed for ITS2. BLAST analysis of ITS2 revealed 100% identity match for multiple species within *Aedes* genus. Phylogenetic analysis of the *ITS2* gene confirmed the genus of the specimen as *Aedes* but did not provide strong bootstrap support for species level identification. For COI, nineteen different haplotypes were identified, sixteen among the Kebridehar samples and five among the Degehabur samples. The *COI* tree confirmed the species as *Ae*. *aegypti* (Fig 6) with strong bootstrap support (bs = 98).

**Fig 7:**
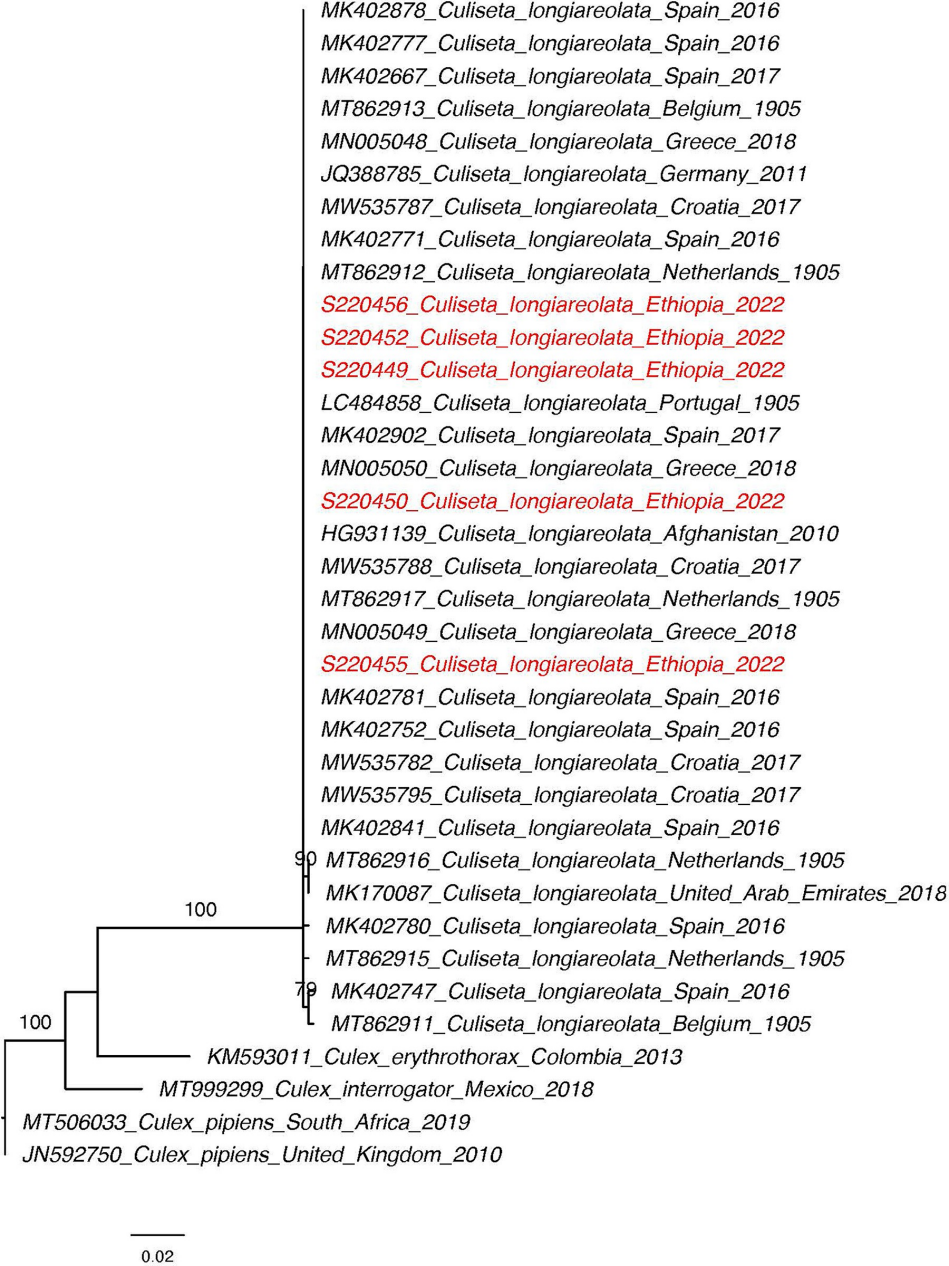
Maximum likelihood phylogenetic tree of *Culiseta longiarelota COI*. Ethiopian sequences are indicated in red. Bootstrap value are based on 1000 replicates. Only bootstraps above 70 are shown.

**Fig 8:**
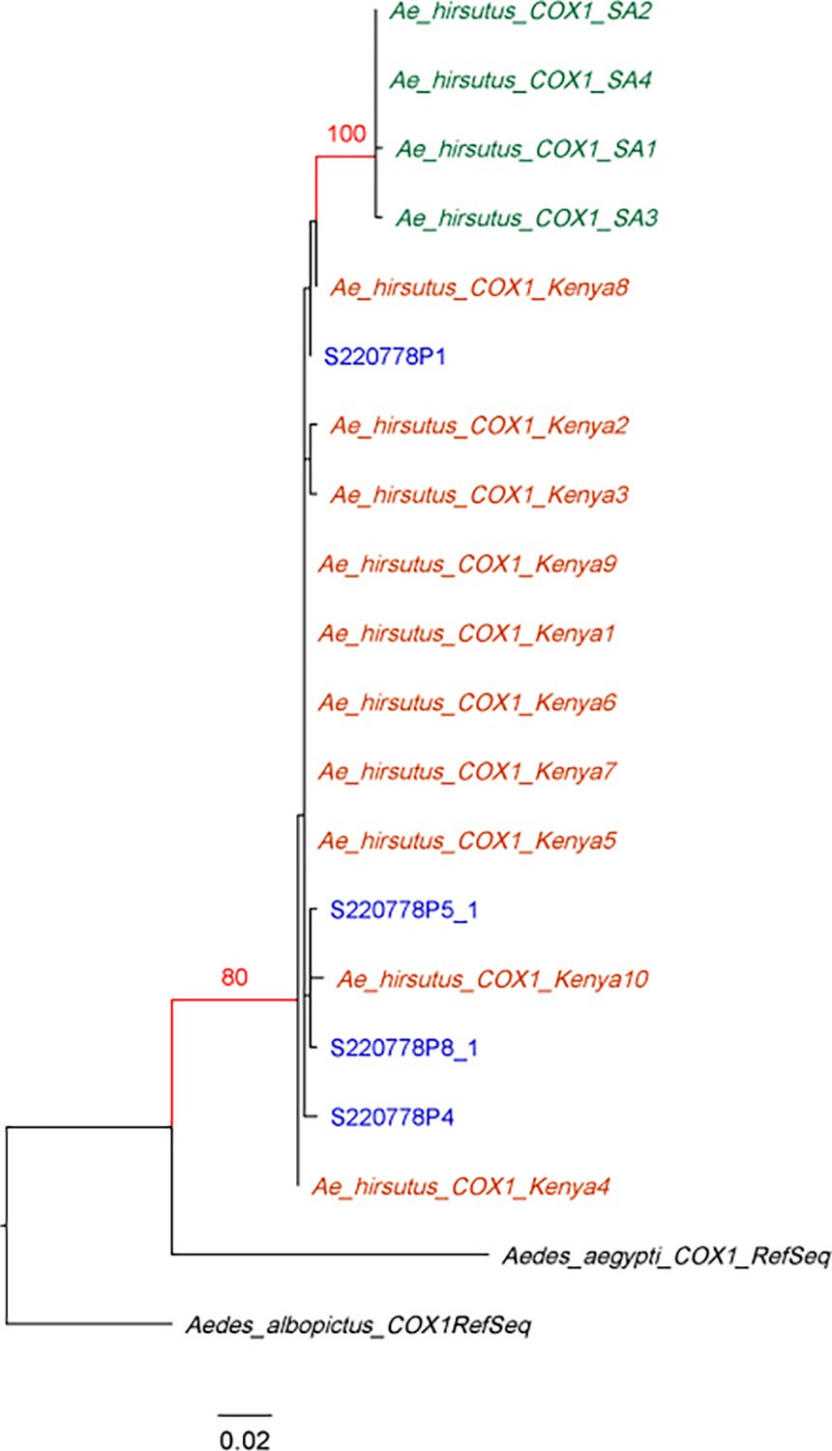
Maximum likelihood phylogenetic tree of *Aedes hirsutus COI*. Ethiopian sequences are indicated in blue. Bootstrap value are based on 1000 replicates. Only bootstraps above 70 are shown.

The unknown specimens collected in Jigjiga in 2022 (S1 and S2 Figs) were genetically analyzed separately. BLAST analysis of COI returned *Culiseta longiareolata* and *Aedes hirsutus*. Phylogenetic analysis of the COI sequences for each species along with database sequences from Genbank confirmed the identity of these specimens (bootstrap = 100 for *Cs*. *longiareolata*, bootstrap = 80 for *Ae*. *hirsutus*) (Figs 7 and 8). *Cs*. *longiareolata* sequences were identical to sequences originating from Europe (Spain, Belgium, Greece, Croatia, Netherlands, and Portugal) and the Middle East (United Arab Emirates and Afghanistan) (Fig 8).

## Discussion

*Aedes aegypti* is considered the main vector of dengue viruses and other arboviral diseases worldwide. Therefore, entomological study was carried out to identify the presence of *Ae*. *aegypti* in urban areas of the Somali region and to study its seasonality and breeding habitats in Kebridehar town. Our data provides a baseline for tracking the transmission of arboviruses in the Somali region. Our study revealed that *Ae*. *aegypti* was documented in all study sites such as Jigjiga, Degehabur, Kebridehar and Godey, it is widely spread across sub-Saharan Africa and Asian countries [15,16]. Similarly, in Ethiopia, this species was detected in urban areas in Dire Dawa [10, 17], Metema, Humera [18] and Kebridehar [13]. The presence of this mosquito in each month surveyed indicates an established *Aedes* mosquito population in Kebridehar. Recent outbreaks of dengue and chikungunya have been reported in Kebridehar and Dire Dawa [13].

Analysis of the mitochondrial COI gene provided a good confirmation of species identification and revealed multiple COI haplotypes exists among the Ethiopian *Ae*. *aegypti*. More genomic data is needed for deeper phylogeographic analysis. While some support for subpopulation differentiation was observed among some Degehabur samples (bootstrap = 84), most *Ae*. *aegypti* in Ethiopia and database sequences representing Kenya, Australia, and Indonesia clustered into a large branch with significant support for distinction from *Ae*. *albopictus*. The high level of variation supports the evidence, *Ae*. *aegypti* abundance in east Ethiopia of a long-established population.

In the phylogenetic analysis, the set of samples from Ethiopia grouped away from the Australian and Indonesian samples with a significant bootstrap value of 73. On the other hand, the Kenyan samples grouped with the Ethiopian samples suggesting a close relationship due to the geographic proximity. Overall, this phylogenetic tree indicates the origin of the *Aedes* population in Ethiopia is more likely to be from a neighboring country. The differentiation trends (no significant bootstrap values) among Ethiopian samples suggest the absence of subpopulations of *Ae*. *aegypti* among our collection in Ethiopia, though further sequencing is needed to be definitive. To further investigate the origin of the Ethiopian *Aedes* populations studied here, we need to explore the level of differentiation between subpopulations within Ethiopia, and we need to compare the level of differentiation within Ethiopia to the differentiation between Ethiopia and neighboring countries.

An essential component of vector competence is an understanding of the magnitude of temporal variability and consistency. We assessed the seasonality of *Ae*. *aegypti* for the first time in Somali region of Ethiopia. A high number of *Ae*. *aegypti* were detected during the wet season, with highest number detected in November, followed by October, and December. Similar studies in West Africa found that adult *Ae*. *aegypti* population numbers often show a positive correlation with rainfall with a single peak in abundance in areas with a single wet season [19, 20]. A high density of *Aedes* mosquito species was observed during the wet season compared to the dry season in Cameroon [21]. However, a study in Ghana showed that *Aedes* populations increased during the dry season rather than the wet season [22]. In our study, the detection during Ethiopia’s dry season may be due to the presence of water storage containers in every house serving as breeding habitats.

The present study showed that *Ae*. *aegypti* larvae and pupae were found in discarded tires, cemented cisterns, plastic containers, and barrels. All the positive water holding containers for *Aedes* mosquito were found outside houses which was 10m radius. In addition, the Larval Indices found in this survey were relatively high. These values were similar to what has been reported in Dire Dawa, Metema and Humera [10, 18]. High Larval Indices were also reported in Kebridehar in 2017 that coincided with an outbreak of dengue fever [13]. The high Larval Indices in Kebridehar in this study suggests a persistent high risk for dengue outbreaks. Good environmental sanitation and a consistent water supply system would reduce the number of putative *Ae*. *aegypti* breeding sites [23] particular in Kebridehar which had the highest larval indices.

In our study, discarded tires contained the most *Aedes* mosquitoes. These discarded tires that were distributed throughout Kebridehar Town were found placed outdoors, stationary for an extended period of time, and filled with water during the rainy season. These factors combined made for ideal mosquito breeding habitats. Our finding highlight discarded tires as a major breeding source is consistent with previous studies conducted in Dire Dawa, Metema and Humera, Ethiopia [10,17,18] and in Tanzania, Zanzibar, and Mozambique [23,24,25]. Collectively, these findings point to the threat of discarded tires and more interventions involving the appropriate handling of old tires should be implemented.

Our study also revealed that *Aedes* were also breeding in cemented cistern water containers. Outdoor cemented cistern water containers (local named as Birka) are present with most houses in Kebridehar Town as water reservoirs in the absence of an efficient water system. This water is brought from a long distance due to the scarcity of water around the town which is a dry area, and people used this water for drinking and daily consumption. The role of these types of larger water containers for *Aedes* breeding and a driver to the movement of *Aedes* was previously reported (i.e., birka) in Dire Dawa city [17]. We observed that majority of cemented cistern water containers those build for drinking purposes were well covered with corrugated iron but the stored water is being kept for more than a month and this favors *Aedes* and other mosquito spp. to breed and spread throughout the town which likely could attribute for high diseases transmission and the possibility to increase outbreaks. Following that, evidence revealed that frequent outbreaks of dengue and chikungunya were investigated in Kebridehar town due to an increase in vectors in the town [13]. More studies are needed to determine how impactful these cisterns are for the spread of *Ae*. *aegypti* in Ethiopia.

Another potential source of *Aedes* larvae and pupae in Kebridehar Town was plastic containers. It is well known that Kebridehar Town is urban and growing and expanding, many house constructions were underway. Many houses were being built, and water was being stored in plastic containers for construction. Because these containers were open, exposed, and static for a long time with poor handling, it is likely that mosquitoes used them to lay their eggs. If larvae and pupae were able to survive and develop into adult mosquitoes, there would be significant disease transmission in the town. In similar studies, *Ae*. *aegypti* was more prevalent in plastic containers used for storing water in Cameron [21]. Therefore, the local government and the regional health bureau must act when there is construction in the town. People and contractors should be aware that when they build plastic containers and other water storage, the water containers need to be covered, kept clean, and replaced right away.

Interestingly, we also identified two species in the Somali Region not previously investigated including *Cs*. *longiarelota* and *Ae*. *hirsutus*. These two species were found in plastic water containers built for construction purposes similarly to what was reported previously [26]. *Cs*. *longiarelota* species has been reported in the Middle East [27] and is invasive to Europe [26]. Cs. longiarelota is reported vector of West Nile virus. While *Ae*. *hirsutus* is not a known as a vector of human diseases, further investigation of this species in needed. Our findings highlight the importance of incorporating molecular identification into *Aedes* surveillance to identify other potential vectors of harmful diseases.

## Conclusion

The study provides preliminary evidence of *Ae*. *aegypti* in all study sites including Jigjiga, Degehabur, Kebridehar and Godey towns. The results show that *Ae*. *aegypti* were found close to human due to the presence of breeding habitat like cemented water reservoir (local name called Birka), tires, plastic containers, and barrels inside human dwelling. The high observed values of the *Aedes* mosquito larval indices suggest a high risk of arbovirus transmission when arboviral cases become established in the area. Therefore, early interventions are necessary to combat the burden of emerging arboviral diseases and it is critical planning and implementation of vector control strategy in the region. Further, *Ae*. *aegypti* control programs should concentrate their interventions on the education and engagement of residents in appropriate use and disposal of old tires and covering of water holding containers.

## Supporting information

S1 Fig Image of *Culiseta longiareolata*. (JPG)

S2 Fig Image of *Aedes hirsutes*. (JPG)
