# Supplementary figures and images for: Breeding habitats, bionomics and phylogenetic analysis of Aedes aegypti and first detection of Culiseta longiareolata, and Ae. hirsutus in Somali Region, eastern Ethiopia
Source: PLoS One. 2024 Jan 2;19(1):e0296406. doi: 10.1371/journal.pone.0296406 (PMC10760653; doi:10.1371/journal.pone.0296406)

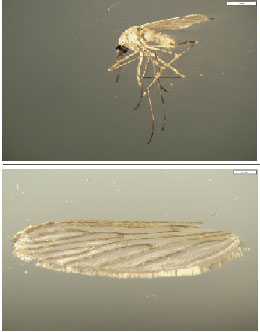

Supplement: S1 Fig — (JPG) [file pone.0296406.s001.jpg]

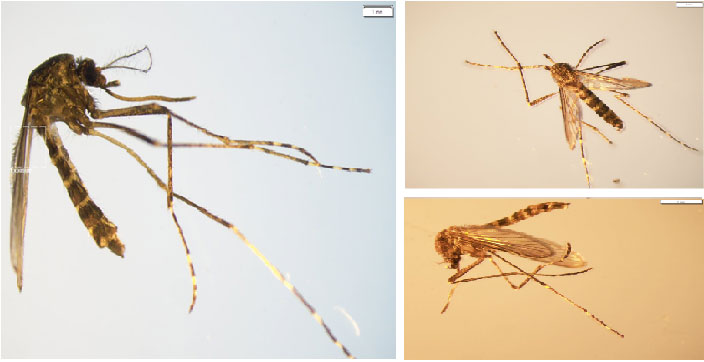

Supplement: S2 Fig — (JPG) [file pone.0296406.s002.jpg]
